# Therapeutic outcome of dapagliflozin in patients with type 2 diabetes and non-alcoholic fatty liver disease: a meta-analysis of randomized controlled trials

**DOI:** 10.4314/ahs.v23i2.48

**Published:** 2023-06

**Authors:** Changlun Hu, Tianhua Qu, Lin LI, Yi Huang, Huabao Liu, Chunyan Rao

**Affiliations:** 1 Department of Internal Medicine, Chongqing Nanan District Traditional Chinese and Western Medicine Hospital, Chongqing, 400061, China; 2 Department of Hepatology, Chongqing Hospital of Traditional Chinese Medicine, Chongqing 400021, China

**Keywords:** Dapagliflozin, type 2 diabetes, non-alcoholic fatty liver disease, randomized controlled trials

## Abstract

**Introduction:**

The efficacy of dapagliflozin remains controversial for patients with type 2 diabetes and non-alcoholic fatty liver disease. We conduct this meta-analysis to explore the influence of dapagliflozin versus placebo on the treatment efficacy of type 2 diabetes complicated with non-alcoholic fatty liver disease.

**Methods:**

We have searched PubMed, EMbase, Web of science, EBSCO, and Cochrane library databases through November 2021 for randomized controlled trials (RCTs) assessing the efficacy of dapagliflozin versus placebo for type 2 diabetes complicated with non-alcoholic fatty liver disease. This meta-analysis is performed using the random-effect model.

**Results:**

Four RCTs are included in the meta-analysis. Overall, compared with patients with type 2 diabetes and non-alcoholic fatty liver disease, dapagliflozin treatment is associated with significantly reduced alanine aminotransferase (ALT, standard mean difference [SMD]=-1.27; 95% confidence interval [CI]span style=“font-family: ‘Times New Roman’”>=-1.60 to -0.95; P<0.00001), aspartate-aminotransferase (AST, SMD=-1.37; 95% CI=-2.08 to -0.65; P=0.0002), fasting glucose (SMD=-0.78; 95% CI=-1.28 to -0.27; P=0.003) and HbA1c (SMD=-0.77; 95% CI=-1.21 to -0.34; P=0.0005), but demonstrated no obvious influence on homeostatic Model Assessment of Insulin Resistance (HOMA-IR, SMD=-0.36; 95% CI=-0.86 to 0.14; P=0.16).

**Conclusions:**

Dapagliflozin benefits to improve hepatic function and glucose control in patients with type 2 diabetes and non-alcoholic fatty liver disease, as evidenced by the reduction in ALT, AST, fasting glucose and HbA1c.

## Introduction

Non-alcoholic fatty liver disease has been reported to have strong association with type 2 diabetes 1-4. Its prevalence reaches up to 50% in patients with type 2 diabetes 5. Non-alcoholic fatty liver disease results in various liver disorders such as hepatocellular steatosis and severe non-alcoholic steatohepatitis, which may progress to hepatic fibrosis and cirrhosis 6-8. However, there are still no any FDA-approved drugs for non-alcoholic fatty liver diseases.

Anti-diabetics drugs have shown some potential in the treatment of non-alcoholic fatty liver disease 9-12. Some anti-diabetic drugs such as sodium-glucose co-transporter (SGLT-2) inhibitors can ameliorate the pathological mechanisms of non-alcoholic fatty liver disease such as body weight, blood pressure, inflammation and insulin resistance 13. As one of the latest oral SGLT-2 inhibitors, dapagliflozin has been commonly prescribed in patients with type 2 diabetes. The anti-hyperglycemic effect of dapagliflozin relies on the inhibition of glucose reabsorption by specifically binding to SGLT-2 in the renal proximal tubule. SGLT-2 inhibitors can also result in weight loss and the reduction in body fat 14. These suggest that dapagliflozin may have beneficial effect on non-alcoholic fatty liver diseases.

However, the benefit of dapagliflozin for non-alcoholic fatty liver disease with concomitant type 2 diabetes has not been well established. Recently, several studies on the topic have been published, and the results are conflicting 13, 15, 16. with accumulating evidence, we therefore perform this meta-analysis of RCTs to explore the efficacy of dapagliflozin versus placebo for patients with non-alcoholic fatty liver disease and type 2 diabetes.

## Materials and methods

Ethical approval and patient consent are not required because this is a meta-analysis of previously published studies. This meta-analysis are conducted and reported in adherence to PRISMA (Preferred Reporting Items for Systematic Reviews and Meta-Analyses) 17, 18.

### Search strategy and study selection

Two investigators have independently searched the following databases (inception to November 2021): PubMed, EMbase, Web of science, EBSCO and Cochrane library databases. The electronic search strategy is conducted using the following keywords: “diabetes” AND “non-alcoholic fatty liver disease” OR “steatohepatitis” AND “dapagliflozin”. We also check the reference lists of the screened full-text studies to identify other potentially eligible trials.

The inclusion criteria are as follows: (i) patients are diagnosed with type 2 diabetes and non-alcoholic fatty liver disease; (ii) intervention treatments are dapagliflozin versus placebo; (iii) study design is RCT.

### Data extraction and outcome measures

We have extracted the following information: author, number of patients, age, female, weight, body mass index and detail methods in each group. Data have been extracted independently by two investigators, and discrepancies are resolved by consensus. The primary outcomes are alanine aminotransferase (ALT), aspartate-aminotransferase (AST). Secondary outcomes include fasting plasma glucose, HbA1c and homeostatic Model Assessment of Insulin Resistance (HOMA-IR).

### Quality assessment in individual studies

Methodological quality of the included studies is independently evaluated using the modified Jadad scale 18, 19. There are three items for Jadad scale: randomization (0-2 points), blinding (0-2 points), dropouts and withdrawals (0-1 points). The score of Jadad Scale varies from 0 to 5 points. An article with Jadad score≤2 is considered to be of low quality. If the Jadad score is ≥3, the study is thought to have high quality 20, 21.

### Statistical analysis

We estimate the standard mean difference (SMD) with 95% confidence interval (CI) for all continuous outcomes. The random-effects model is used regardless of heterogeneity. Heterogeneity is reported using the I2 statistic, and I2 > 50% indicates significant heterogeneity 22. Whenever significant heterogeneity is present, we search for potential sources of heterogeneity via omitting one study in turn for the meta-analysis or performing subgroup analysis. All statistical analyses are performed using Review Manager Version 5.3 (The Cochrane Collaboration, Software Update, Oxford, UK).

## Results

### Literature search, study characteristics and quality assessment

**Figure 1:**
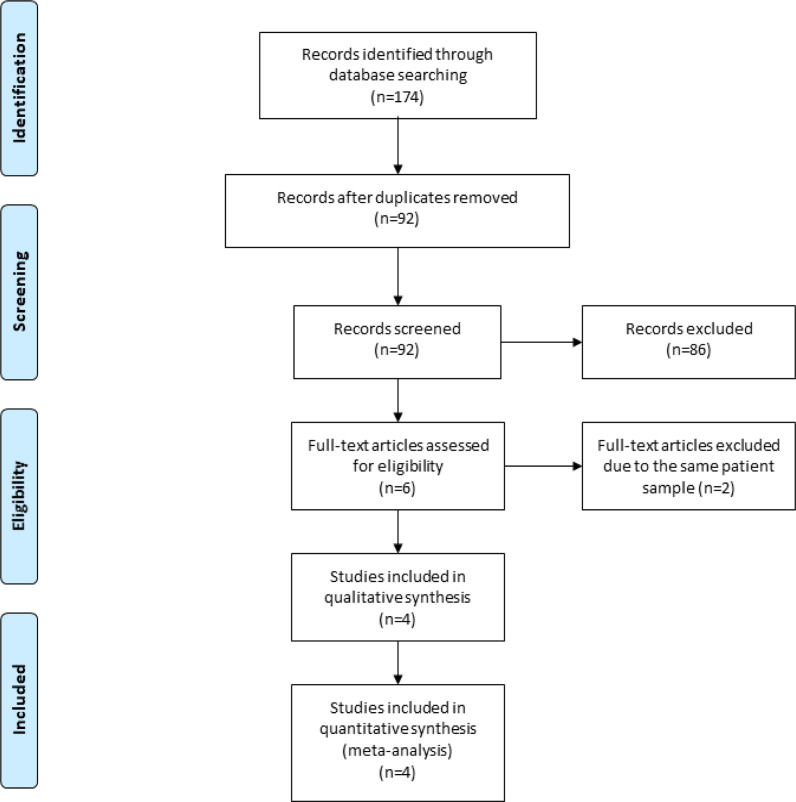
Flow diagram of study searching and selection process

A detailed flowchart of the search and selection results is shown in Figure 1. 174 potentially relevant articles are identified initially. Finally, four RCTs that meet our inclusion criteria are included in the meta-analysis 13, 15, 16, 23.

The baseline characteristics of four eligible RCTs in the meta-analysis are summarized in Table 1. The four studies are published between 2018 and 2021, and total sample size is 275. The doses of dapagliflozin range from 5 mg to 10 mg daily, while the treatment durations are 12 weeks or 24 weeks. Three studies report the same patient sample 16, 24, 25, and two of them are excluded 24, 25.

**Table 1:** Characteristics of included studies

NO.	Author	Dapagliflozin group						Control group						Jadad scores
		Number	Age (years)	Female (n)	Weight (kg)	Body mass index (kg/m2)	Methods	Number	Age (years)	Female (n)	Weight (kg)	Body mass index (kg/m2)	Methods	
1	Aso 2019	33	56.2 ±11.5	14	73.6(61.9, 80.8)	27.6±4.7	dapagliflozin (5 mg/day) for 24 weeks	24	57.1 ±13.8	9	74.9(65.6,81.6)	28.7±3.5	placebo	3
2	Eriksson 2018	21	65.0±6.5	5	90.2±8.7	30.5±2.8	dapagliflozin (10 mg/day) for 12 weeks	21	65.6±6.1	4	93.0±12.2	30.3±3.1	placebo	4
3	Hussain 2021	67	29 ± 16	20	90 ± 13.5	29.5 ± 2.5	dapagliflozin (510 mg/day) for 12 weeks	71	31 ± 14	19	85 ± 17.8	31.5 ± 3.0	placebo	5
4	Phrueksotsai 2021	18	57.0 ± 6.9	13	73.8 ± 12.7	29.6 ± 4.0	dapagliflozin (10 mg/day) for 12 weeks	20	61.2 ± 7.2	13	71.0 ± 11.3	28.8 ± 4.1	placebo	4

Data are mean ± SD or median and inter-quartile ranges.

Among the four studies included here, two studies report ALT, AST and fasting glucose 13, 15, while three studies report HbA1c and HOMA-IR 13, 15, 23. Jadad scores of the four included studies vary from 3 to 5, and all four studies have high quality according to quality assessment.

### Primary outcomes: ALT and AST

**Figure 2:**
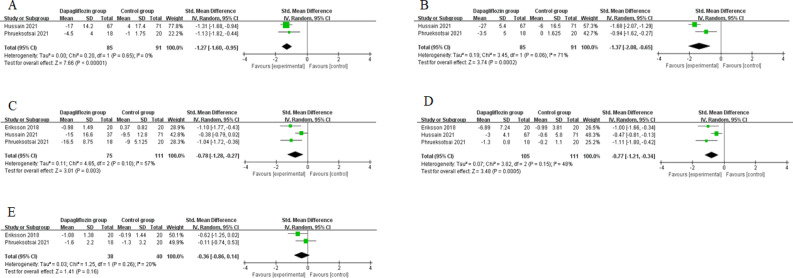
Forest plot for the meta-analysis of ALT (A), AST (B), fasting glucose (C), HbA1c (D), and HOMA-IR (E)

These outcome data are analysed with the random-effects model, and compared to control group for type 2 diabetes complicated with non-alcoholic fatty liver disease, dapagliflozin treatment is associated with significantly reduced ALT (SMD=-1.27; 95% CI=-1.60 to -0.95; P<0.00001) with no heterogeneity among the studies (I2=0%, heterogeneity P=0.65, Figure 2A), and AST (SMD=-1.37; 95% CI=-2.08 to -0.65; P=0.0002) with significant heterogeneity among the studies (I2=71%, heterogeneity P=0.06, Figure 2B).

### Sensitivity analysis

Significant heterogeneity is observed among the included studies for the AST. Because there are only two included studies, we do not perform the sensitivity analysis by omitting one study in turn.

### Secondary outcomes

In comparison with control group for type 2 diabetes complicated with non-alcoholic fatty liver disease, dapagliflozin treatment can substantially decrease fasting glucose (SMD=-0.78; 95% CI=-1.28 to -0.27; P=0.003; Figure 2C) and HbA1c (SMD=-0.77; 95% CI=-1.21 to -0.34; P=0.0005; Figure 2D), but unravel no obvious influence on HOMA-IR (SMD=-0.36; 95% CI=-0.86 to 0.14; P=0.16; Figure 2E).

## Discussion

The treatment of type 2 diabetes with concomitant non-alcoholic fatty liver disease is still challenging and dapagliflozin has obtained an increasing attention. Our meta-analysis included four RCTs and 275 patients with type 2 diabetes and non-alcoholic fatty liver disease. The results found that dapagliflozin treatment can substantially improve the hepatic function as evidenced by the reduced ALT and AST, and also showed the beneficial effect on glycemic control as shown by the decreased fasting glucose and HbA1c.

Inhibition of inflammatory and oxidative stress in patients with non-alcoholic fatty liver disease plays an important role in reducing the rate of progression and the risk of cardiovascular disorders^26^-^28^. In addition to glycemic control, dapagliflozin shows the favourable influence on body weight, blood pressure, insulin resistance, silent inflammation, oxidative stress and hyperuricemia. Dapagliflozin has the ability to decrease body weight, which is usually accepted as the first-line nonpharmacological management in non-alcoholic fatty liver disease. The reduction in blood weight and glucose by dapagliflozin ameliorates insulin resistance, which is the main metabolic abnormalities in patients with non-alcoholic fatty liver disease.

Regarding the sensitivity analysis, significant heterogeneity remained for AST, but we can not perform the sensitivity analysis by omitting one study in turn due to only two included RCTs. Several factors may result in the heterogeneity. Firstly, the doses of dapagliflozin are various, ranging from 5 mg to 10 mg daily. Secondly, the combination drugs with dapagliflozin are different, which may affect the efficacy assessment of dapagliflozin. Thirdly, the severity levels of non-alcoholic fatty liver disease are different, which may affect the response of dapagliflozin. Our meta-analysis also has some important limitations. Firstly, our analysis is based on four RCTs, and three of them have a relatively small sample size (n<100). Overestimation of the treatment effect is more likely in smaller trials compared with larger samples. Secondly, there is significant heterogeneity, which may be caused by different doses and combination methods of dapagliflozin. Thirdly, different etiologies and severity levels of non-alcoholic fatty liver disease in patient with type 2 diabetes may affect the efficacy assessment of dapagliflozin.

## Conclusions

Dapagliflozin provides additional benefits to improve hepatic function and glucose control for patients with type 2 diabetes and non-alcoholic fatty liver disease, as shown by the by the reduction in ALT, AST, fasting glucose and HbA1c.
